# The Construction of Phosphorus-Doped g-C_3_N_4_/Rh-Doped SrTiO_3_ with Type-II Band Alignment for Efficient Photocatalytic Hydrogen Evolution

**DOI:** 10.3390/nano12244428

**Published:** 2022-12-12

**Authors:** Bin Wang, Peng Li, Hanjing Hao, Huijie He, Hairui Cai, Fanfan Shang, Bei An, Xiaoqian Li, Shengchun Yang

**Affiliations:** 1 MOE Key Laboratory for Non-Equilibrium Synthesis and Modulation of Condensed Matter, Key Laboratory of Shaanxi for Advanced Materials and Mesoscopic Physics, State Key Laboratory for Mechanical Behavior of Materials, School of Physics, Xi’an Jiaotong University, No. 28 West Xianning Road, Xi’an 710049, China; 2 National Innovation Platform (Center) for Industry-Education Integration of Energy Storage Technology, Xi’an Jiaotong University, No. 28 West Xianning Road, Xi’an 710049, China; 3 Shaanxi Collaborative Innovation Center for Hydrogen Fuel Cell Performance Improvement, Xi’an Jiaotong University, No. 28 West Xianning Road, Xi’an 710049, China

**Keywords:** type-II heterojunction, photocatalyst, photocatalytic hydrogen evolution, charge separation

## Abstract

It is of great importance to promote charge separation in photocatalysts for enhanced photocatalytic activity under visible light irradiation. In this work, a type-II heterostructured photocatalyst was constructed by compositing phosphorus-doped g-C_3_N_4_ (P-CN) and Rh-doped SrTiO_3_ (Rh-STO) via a thermal calcination treatment. A series of characterizations were conducted to investigate the structure of heterostructured P-CN/Rh-STO. It was found that Rh-STO interacted with in situ generated P atoms from the decomposition of P-CN during the calcination process, thus leading to the formation of heterojunction of P-CN/Rh-STO. Compared with the single component, i.e., P-CN or Rh-STO, the obtained P-CN/Rh-STO showed superior photocatalytic activity to that of both P-CN and Rh-STO due to the effective charge separation across the heterojunction between P-CN and Rh-STO.

## 1. Introduction

With the rapid development of the economy, it has become more and more urgent to address the energy crisis and environmental pollution [1,2,3,4]. As a promising technology, photocatalytic water splitting can utilize solar energy to produce sustainable hydrogen from water, which has drawn tremendous attention in recent decades [5,6,7,8]. The semiconducting photocatalysts play a core role during the photocatalytic process, including light absorption, charge separation, and surface redox reactions [9,10]. Thus, it is the key to developing efficient photocatalysts for enhanced photocatalytic performance.

Since the pioneering work by Wang et al. in 2009 [11], g-C_3_N_4_, a polymeric semiconducting material with heptazine ring structures, exhibited several advantages for photocatalysis, such as excellent physiochemistry stability, earth-abundant resource, suitable band structure, etc. [12]. Various precursors, such as melamine, cyanamide, dicyanamide, urea, and thiourea, have been used for synthesizing g-C_3_N_4_ [7]. However, g-C_3_N_4_ still suffers from limited visible light absorption and severe charge combination during the photocatalytic process, which greatly restricts its photocatalytic performance. Up to now, many efforts, such as morphology engineering [13,14] and introducing vacancy [15], etc., have been made to address the above shortcomings of g-C_3_N_4_ [2,16,17,18]. It has been demonstrated that metal element doping can effectively extend the absorption of g-C_3_N_4_ in the visible light region [19,20]. For example, Cai et al. found that introducing Co atoms into the centers of adjacent heptazine rings in g-C_3_N_4_ can not only increase the visible light absorption of g-C_3_N_4_, but also act as the rapid electron transfer channel during the photocatalytic process [21]. Wang et al. reported that the doping of Cu atoms into g-C_3_N_4_ can regulate its energy band structure for enhanced its visible light absorption, and improve the separation and transfer of photogenerated charges [22]. Apart from metal elements, various nonmetal elements, such as C, N, S, P, I, etc. have also been introduced into g-C_3_N_4_ [7,23]. Zhu et al. designed phosphorus-doped g-C_3_N_4_ with nanostructured flowers via a co-condensation method. The authors demonstrated that both the flower-like structures and phosphorus doping promoted light trapping, mass transfer, and charge separation, thus greatly increasing its catalytic hydrogen evolution under visible light irradiation [24].

Different from element doping, it has been demonstrated that constructing heterojunctions with type-II band structure alignment is also an effective strategy to improve charge separation during the photocatalysis process [25,26,27]. For example, Zhong et al. prepared covalently bonded 2D/2D O-g-C_3_N_4_/TiO_2_ heterojunction for improved visible-light photocatalytic hydrogen evolution. In such a heterostructured photocatalyst, N-O-Ti covalent bonding led to the strong affinity between TiO_2_ and O-g-C_3_N_4_ 2D structures, thus boosting the visible-light-driven activity for hydrogen evolution [28]. Cai et al. designed a type II heterojunction by compositing TiO_2_ and g-C_3_N_4_ with an oriented charge transfer path, which can effectively separate the photo-generated charge carriers, thus leading to the greatly enhanced photocatalytic hydrogen performance [29].

Apart from TiO_2_, the perovskite-structured SrTiO_3_ [30], which is a promising photocatalyst due to its high physical and chemical stability, non-toxicity, and low cost [30,31], is also used for constructing g-C_3_N_4_ based heterostructured photocatalyst [32,33]. For example, Pan et al. designed a unique MoS_2_-lamellas-modified core-shell structured C_3_N_4_/SrTiO_3_ via a continuous hydrothermal-annealing method [32], and found that the core-shell heterojunction can promote the separation of photo-generated charge carriers efficiently. However, the SrTiO_3_ with a bandgap of 3.2 eV in such heterostructured system cannot utilize visible light for photocatalytic hydrogen evolution.

It has been reported that introducing dopants, such as Cr [34], Rh [35], etc., can reduce the bandgap of SrTiO_3_ for promoted photocatalytic activity. Considering the drawback of non-visible light response for SrTiO_3_ and limited light absorption of g-C_3_N_4_, in this work, a type-II heterostructured photocatalyst was constructed by compositing phosphorus-doped g-C_3_N_4_ (P-CN) and Rh-doped SrTiO_3_ (Rh-STO) via a thermal calcination treatment. It was found that Rh-STO interacted with P element, which resulted from the decomposition of P-CN during the calcination process, thus leading to the formation of heterojunction of P-CN/Rh-STO. A series of characterizations were conducted to investigate the structure of heterostructured P-CN/Rh-STO. The obtained P-CN/Rh-STO showed superior photocatalytic activity than that of both P-CN and Rh-STO due to the effective charge separation across the interface between P-CN and Rh-STO.

## 2. Materials and Methods

### 2.1. Chemicals and Materials

Strontium chloride hexahydrate (SrCl_2_·6H_2_O), titanium tetrachloride (TiCl_4_), rhodium trichloride (RhCl_3_), lithium hydroxide monohydrate (LiOH·H_2_O), melamine (C_3_H_6_N_6_), phytic acid (C_6_H_18_O_24_P_6_, IP6), and triethanolamine ((HOCH_2_CH_2_)_3_N, TEOA) were purchased from Sinopharm Chemical Reagent Co., Ltd., Shanghai, China. Hexachloroplatinic acid (H_2_PtCl_6_) was offered by Alfa Aesar, A Johnson Matthey Company. Nafion solution (5%) was purchased from Sigma-Aldrich (Shanghai) Co., Ltd., Shanghai, China. All the reagents were standard analytical grade without further purification. The water used in all syntheses was deionized water with a resistivity of 18.25 MΩ·cm.

### 2.2. Synthesis of Rh-SrTiO_3_

Rh-SrTiO_3_ was prepared by a simple hydrothermal method. Briefly, 0.53 g SrCl_2_·6H_2_O was dissolved into 30 mL of deionized water with stirring for 10 min. Then, 0.21 mL TiCl_4_ was added into the solution with continuous stirring. Then, 30 min later, 0.62 mL of RhCl_3_ solution (the concentration of Rh in the solution is 10 mg/mL) was added. After 30 min, 40 mL 0.5 M LiOH solution was added into the above solution drop by drop. Finally, the evenly stirred reaction solution was transferred into a 100 mL Teflon-lined stainless steel autoclave, followed by heat treatment at 180 °C for 24 h. After cooling, the prepared sample was centrifuged and washed with deionized water several times to remove the residual LiOH. After drying overnight at 60 °C in a vacuum drying oven, the finally obtained sample was ground into powder and named Rh-STO.

### 2.3. Synthesis of P-CN

First, 3 g of melamine and 3.6 g of phytic acid were dispersed in 35 mL of deionized water and stirred vigorously for 1 h under an 80 °C water bath to form an emulsion. Then, the obtained emulsion was transferred to a 50 mL Teflon-lined autoclave and heated at 180 °C for 12 h. After cooling, the sample was centrifuged with deionized water and washed several times to remove the remaining phytic acid. After drying in a vacuum oven at 60 °C overnight, 3 g of the above precursor was put into an alumina crucible with a cover, and placed in a muffle furnace at 550 °C for 4 h in air with a heating rate is 5 °C/min. After cooling, the obtained sample was ground into powder and named as P-CN.

### 2.4. Synthesis of P-CN/Rh-STO

The heterostructured P-CN/Rh-STO-20 was synthesized by a simple calcination process. Briefly, 20 mg Rh-STO, 100 mg P-CN, and 500 μL deionized water were added into a mortar, and ground evenly until the water evaporated completely. Finally, the obtained powder was put into a crucible, wrapped with aluminum foil, and calcined at 450 °C in the air atmosphere for 2 h with a heating rate of 2 °C/min. The finally synthesized powder was named P-CN/Rh-STO-20. To optimize the content of Rh-STO in heterostructured P-CN/Rh-STO-20, various amounts of Rh-STO (0 mg, 10 mg, 50 mg, and 100 mg) were used for synthesizing a series of P-CN/Rh-STO samples via the same method, and the corresponding samples were named P-CN/Rh-STO-0, P-CN/Rh-STO-10, P-CN/Rh-STO-50, and P-CN/Rh-STO-100, respectively.

### 2.5. Photocatalytic Activity Measurements

In a typical experiment of water splitting for hydrogen evolution, 12.5 mg of photocatalyst powder was dispersed into 80 mL containing 10 vol% TEOA solution by sonication for 60 min in a side-irradiation Pyrex cell. After being evacuated by N_2_ gas, the suspension was irradiated by full-wave light or visible light through a cutoff filter (λ ≥ 400 nm) from a 300 W Xe lamp for 4 h. During the light irradiation, the evolved H_2_ gas was collected at the given time intervals and analyzed by gas chromatography equipped with a thermal conductive detector (TCD) and high-purity N_2_ carrier gas.

## 3. Results and Discussions

**Figure 1:**
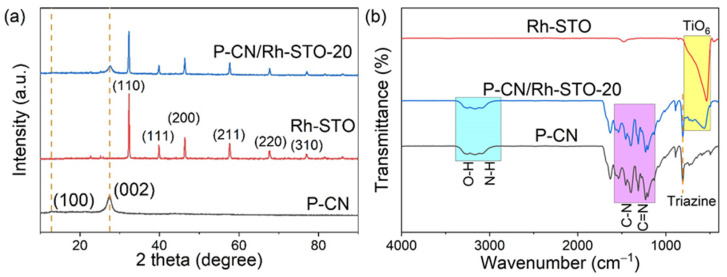
(**a**) XRD patterns and (**b**) FT-IR spectra of P-CN, Rh-STO, and P-CN/Rh-STO-20, respectively.

The XRD patterns of P-CN, Rh-STO, and P-CN/Rh-STO-20 were shown in Figure 1. It can be seen that P-CN showed obvious characteristic peaks at 12.8° and 27.4°, corresponding to (100) and (002) crystal planes of g-C_3_N_4_ [36], respectively, indicating that the introduction of P element did not destroy the basic structure of g-C_3_N_4_. The diffraction patterns of Rh-STO were completely consistent with the standard pattern of cubic perovskite structured SrTiO_3_ (JCPDS No. 00-035-0734, space group: $Pm\bar{3}m$) [37]. In addition to the obvious Rh-STO XRD peaks, P-CN/Rh-STO-20 also exhibited the diffraction peak of (002) plane of P-CN, revealing the successful synthesis of heterostructured P-CN/Rh-STO-20. No obvious diffraction peak of (100) plane of P-CN was observed in the XRD pattern of P-CN/Rh-STO-20, because the strong peak intensities of Rh-STO submerged the (100) peak of P-CN. Figure 1b shows the FT-IR spectra of P-CN, Rh-STO, and P-CN/Rh-STO-20. For the P-CN, the peaks at 805 cm^−1^ can be assigned to the breathing vibration of triazine units [38], the multiple peaks at 1200–1650 cm^−1^ were attributed to the C-N bond and C=N bond [39], and wide peaks at 2900–3400 cm^−1^ corresponded to the O-H bond and N-H bond [40]. The wide absorption peak at 500–800 cm^−1^ for Rh-STO belonged to the stretching vibration of TiO6 octahedron [41]. P-CN/Rh-STO-20 exhibited all the characteristic peaks of P-CN and Rh-STO, indicating the existence of both P-CN and Rh-STO in heterostructured P-CN/Rh-STO-20.

**Figure 2:**
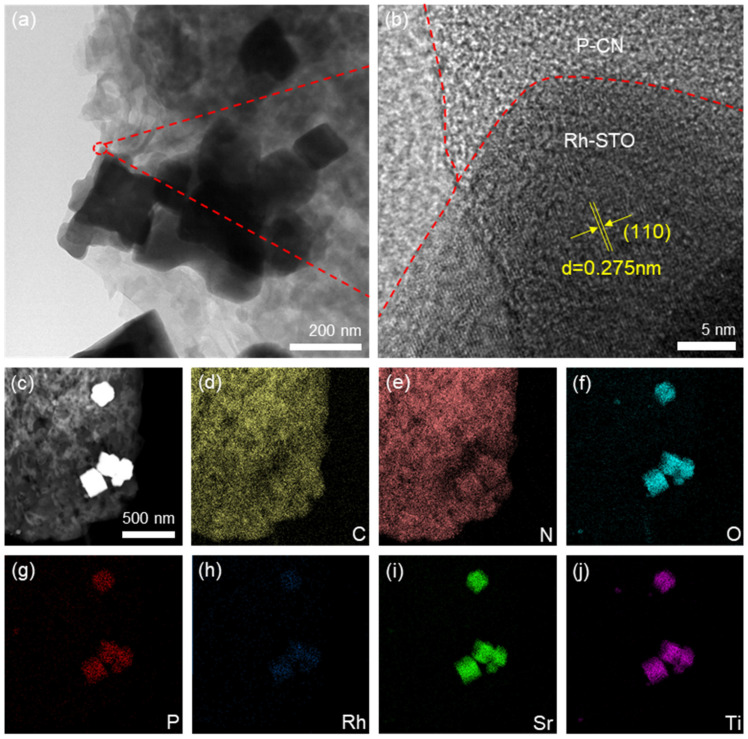
(**a**) TEM image, (**b**) HRTEM image, and (**c**) HAADF-STEM image of P-CN/Rh-STO-20; (**d**–**j**) the C, N, O, P, Rh, Sr, and Ti element mapping of P-CN/Rh-STO-20, respectively.

TEM characterization was conducted to investigate the morphology and structure of heterostructured P-CN/Rh-STO-20. As shown in Figure 2a, P-CN with low contrast presented a typical stacked lamellar structure and the nanoparticles with high contrast were Rh-STO. It can be seen that Rh-STO was attached closely to the surface of P-CN, confirming the successful construction of P-CN and Rh-STO. Figure 2b shows the HRTEM image of P-CN/Rh-STO-20, which is recorded from the red dotted circle in Figure 2a. The dotted red lines were the boundary between carbon film, P-CN, and Rh-STO-20, and the lattice spacing of 0.275 nm can be assigned to the (110) plane of SrTiO_3_ [42]. The elemental mappings of P-CN/Rh-STO-20 in Figure 2d–j were recorded from the STEM image in Figure 2c. It is clear that C and N and P elements were distributed throughout the area of P-CN, and the O, Rh, Sr, and Ti elements coincided with the area of Rh-STO nanoparticles, revealing the successful construction of heterostructured P-CN/Rh-STO. In addition, the P element was also found enriched in the area of Rh-STO nanoparticles. This may be due to the interaction of P from P-CN between Rh-STO during the calcination, thus resulting in the close contact between the two components of heterostructured P-CN/Rh-STO, which would be conducive to the charge transfer across the heterojunction.

To further explore the enrichment of P element on the interface between Rh-STO and P-CN, a series of thermogravimetric characterization was conducted. It can be found in Figure S1 that Rh-STO was thermal stable in the test temperature range (25–800 °C) without decomposition, while the P-CN started to decompose when the temperature higher than 550 °C, and completely decomposed at 688 °C (Figure S2). For the sample of P-CN/Rh-STO, it was clear that P-CN was decomposed completely when the temperature exceeds 630 °C (Figure S3). Thus, the remaining residue was Rh-STO-20 with a mass fraction of 21.11% and the mass fraction of P-CN in P-CN/Rh-STO was 78.89%. As described in the experimental section, the mass ratio of P-CN to Rh-STO was 100:20. Thus, the mass percentages of P-CN and Rh-STO in the mixture before calcination were 16.67% and 83.33%, respectively. Therefore, it can be induced that part of P-CN was decomposed during the calcination process to construct the P-CN/Rh-STO heterojunction. At the same time, ICP-MS was conducted to characterize the content of P in P-CN and P-CN/Rh-STO-20, respectively, as shown in Table S1. It can be seen that the content of P element in P-CN/Rh-STO-20 was 1.88 wt%. Since the mass fraction of P-CN in the heterojunction was 78.89%, the mass fraction of P element relative to P-CN in the heterojunction was 2.38%, while in the pristine P-CN, the content of P element was only 2.10 wt%. Combined with TG analysis, it can be speculated that part of P-CN was decomposed during the calcination process, and the P element should be interacted with Rh-STO, thus increasing the P content relative to P-CN in P-CN/Rh-STO heterojunction. Therefore, P element was enriched at the interface between P-CN and Rh-STO, which was confirmed by the elemental mapping characterization in Figure 2.

**Figure 3:**
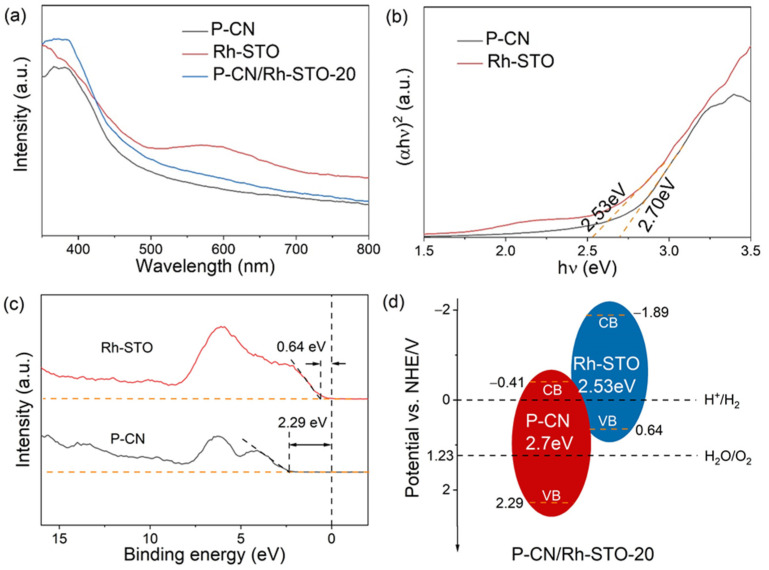
(**a**) UV–Vis absorption spectra of P-CN, Rh-STO, and P-CN/Rh-STO-20; (**b**) the tauc curves obtained from (**a**); (**c**) valence band XPS spectra of P-CN, Rh-STO; (**d**) energy band diagram of P-CN/Rh-STO-20.

Figure 3a shows the UV–Vis absorption spectra of P-CN, Rh-STO, and P-CN/Rh-STO-20, respectively. It was clear that P-CN possessed an absorption edge of ca. 460 nm, while Rh-STO showed an absorption edge of ca. 490 nm. In addition, an obvious absorption appeared near 580 nm for Rh-STO, which should be ascribed to the doping energy level induced by the heteroatoms of Rh in Rh-STO [43,44]. In contrast to P-CN, the heterostructured P-CN/Rh-STO-20 showed a stronger visible light absorption property. Figure 3b shows the tauc plots for P-CN and Rh-STO, respectively, which were derived from the data in Figure 3a. The band gaps of p-CN and Rh-STO were estimated to be ca. 2.70 eV and 2.53 eV, respectively. Moreover, the valence band XPS spectra in Figure 3c revealed that the valence band edge potentials of P-CN and Rh-STO were located at ca. 2.29 V and 0.64 V (vs. NHE), respectively. Combined with the calculated bandgaps from Figure 3b, the potential of the conduction band edge for P-CN and Rh-STO was ca. −0.41 V and −1.89 V (vs. NHE), respectively. Therefore, it can be inferred that the heterostructured P-CN/Rh-STO-20 possessed a type II band alignment structure, as shown in Figure 3d.

**Figure 4:**
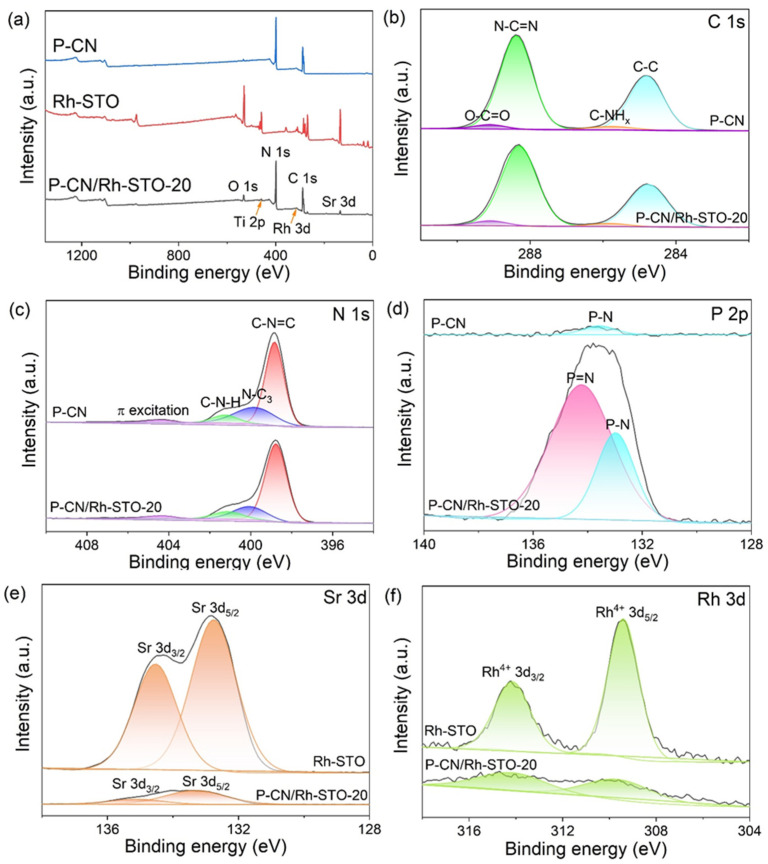
(**a**) XPS survey spectra of P-CN, Rh-STO, and P-CN/Rh-STO-20; (**b**) C 1s XPS spectra, (**c**) N 1s XPS spectra, and (**d**) P 2p XPS spectra of P-CN and P-CN/Rh-STO-20; (**e**) Sr 3d XPS spectra and (**f**) Rh 3d XPS spectra of Rh-STO and P-CN/Rh-STO-20, respectively.

Figure 4 shows the XPS spectra of P-CN, Rh-STO, and P-CN/Rh-STO-20, respectively. As shown in the XPS survey spectra in Figure 4a, the signals of C and N can be detected for P-CN, and the signals of Rh, Sr, Ti and O were detected for Rh-STO. All the signals of C, N, Rh, Sr, Ti, and O were detected for P-CN/Rh-STO-20. No obvious XPS peaks for P in the survey XPS spectra of P-CN and P-CN/Rh-STO were observed, which was due to the weak intensity of P element signal. After the deconvolution of C 1s spectrum for both of P-CN and P-CN/Rh-STO-20 (Figure 4b), four characteristic peaks, which were located at 284.8, 285.9, 288.4, and 289.1 eV, respectively, can be assigned to the C-C bond [45], C-NHx group [46], and N-C=N bond [47] in triazine ring and O=C-C bond on the catalyst surface [48], demonstrating the tri-s-triazine structure of g-C_3_N_4_ were preserved in both P-CN and P-CN/Rh-STO. In the N 1s XPS spectra in Figure 4c, the deconvolution of P-CN and P-CN/Rh-STO-20 showed four characteristic peaks located at 398.8, 399.8, 401.3, and 404.4 eV, respectively, which belonged to C-N=C bond [49], N-C3 group [50], C-N-H group [51], and π excitation [52] in triazine ring, proving the tri-s-triazine structure of g-C_3_N_4_ were maintained in both P-CN and P-CN/Rh-STO-20. In the P 2p spectra (Figure 4d), a small peak corresponding to P-N bond at ca. 133.5 eV was observed for the P-CN, while the XPS peak of P for P-CN/Rh-STO can be deconvoluted into two characteristic peaks at 133.1 and 134.2 eV, which were originated from the P-N bond and P=N bond, respectively [53]. The higher intensity of P signal for P-CN/Rh-STO-20 than that for P-CN may be due to the enrichment of P element at the interface between P-CN and Rh-STO, which was revealed by the ICP-MS in Table S1. Compared with the peak positions of Sr XPS for Rh-STO, the peak positions of Sr for P-CN/Rh-STO were shifted to higher binding energy, which can be ascribed to the compact contact interaction at the interface between P-CN and Rh-STO (Figure 4e). Figure 4f shows the high-resolution XPS spectrum of Rh 3d for Rh-STO and P-CN/Rh-STO. Two characteristic XPS peaks located at 309.4 and 314.1 eV were observed for both of these two samples, which were associated with Rh^4+^ 3d_5/2_ and Rh^4+^ 3d_3/2_, respectively [54].

**Figure 5:**
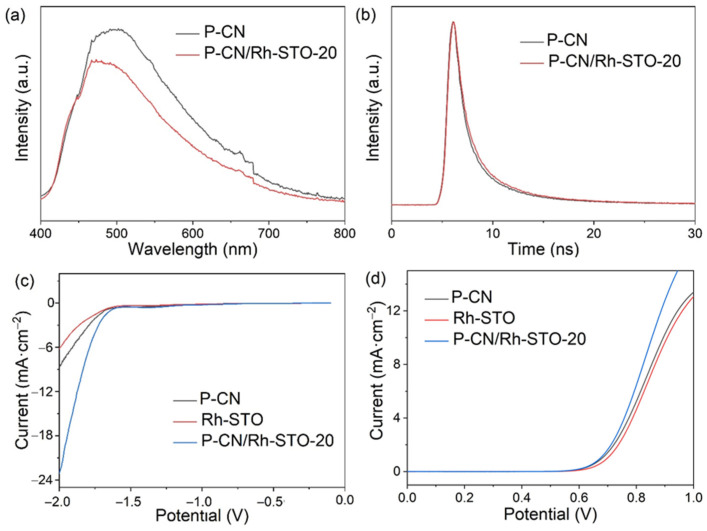
(**a**) Steady-state photoluminescence spectra and (**b**) time-resolved photoluminescence decay spectra of P-CN and P-CN/Rh-STO-20; Linear voltammetric scanning curve of (**c**) electrocatalytic hydrogen evolution reaction and (**d**) electrocatalytic triethanolamine oxidation over P-CN, Rh-STO, and P-CN/Rh-STO-20, respectively.

Figure 5a shows the steady-state photoluminescence spectra of P-CN and P-CN/Rh-STO-20. Compared with P-CN, the fluorescence intensity of P-CN/Rh-STO-20 was significantly reduced, indicating that the heterojunction between P-CN and Rh-STO can effectively reduce the recombination of photogenerated carriers in P-CN/Rh-STO-20. The time-resolved transient photoluminescence decay spectra in Figure 5b revealed that P-CN/Rh-STO-20 possessed a longer lifetime of photogenerated carriers (5.68 ns, Table S2) than that of P-CN (5.22 ns, Table S2), indicating that P-CN/Rh-STO-20 with type II band alignment structure can improve the separation efficiency of charge carriers. We further conducted a series of electrochemistry characterization over P-CN, Rh-STO, and P-CN/Rh-STO-20. Figure 5c,d showed the linear sweep voltammetry scanning curves of P-CN, Rh-STO, and P-CN/Rh-STO-20 toward electrocatalytic hydrogen evolution reaction (HER) and electrocatalytic triethanolamine (TEOA) oxidation reaction, respectively. It was clear that P-CN/Rh-STO-20 exhibited the smallest over potentials among the three samples, revealing that P-CN/Rh-STO-20 can effectively promote the processes of HER and the oxidation of TEOA.

**Figure 6:**
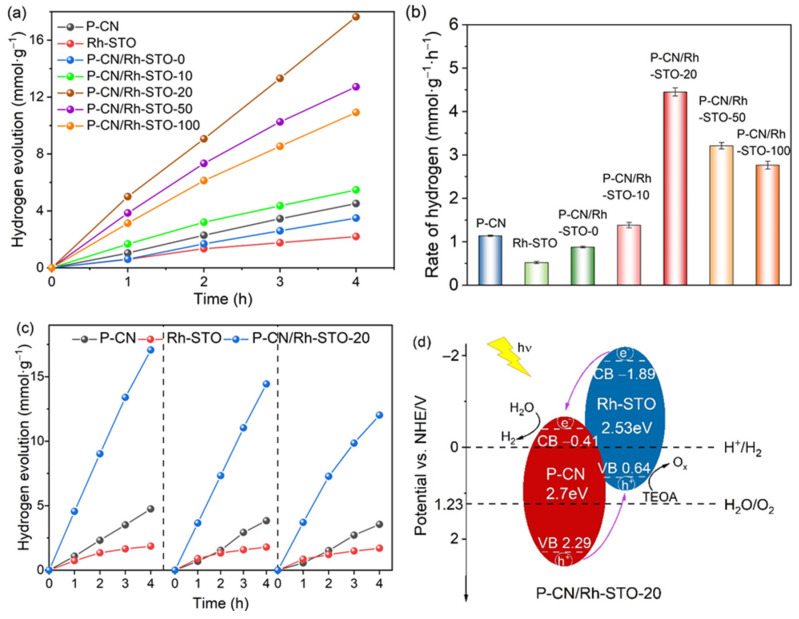
(**a**) Photocatalytic H_2_ evolution over P-CN, Rh-STO and the series of P-CN/Rh-STO samples (1 wt% Pt was used as cocatalyst); (**b**) the rate of hydrogen production of P-CN, Rh-STO, and the series of P-CN/Rh-STO samples; (**c**) cycle experiments of the photocatalytic H_2_ generation over P-CN, Rh-STO, and P-CN/Rh-STO-20; (**d**) schematic diagram of charge transfer in P-CN/Rh-STO-20.

To evaluate the photocatalytic activities of P-CN, Rh-STO, and the constructed P-CN/Rh-STO, the photocatalytic hydrogen evolution experiments over these samples were conducted under visible light irradiation. Typically, the reaction was placed in a side-irradiation Pyrex cell with 80 mL 10 vol% TEOA solution containing the samples. The light was introduced by a 300 W Xe lamp coupled with a 400 nm cutoff filter. In addition to the heterostructured P-CN/Rh-STO-20, a series of P-CN/Rh-STO heterojunction, i.e., P-CN/Rh-STO-0, P-CN/Rh-STO-10, P-CN/Rh-STO-50, and P-CN/Rh-STO-100 were also synthesized by adjusting the mass ratios of P-CN and Rh-STO for the photocatalytic tests. It can be found that the hydrogen production rate of P-CN/Rh-STO-0 was slower than that of P-CN, indicating that the calcination process cannot improve the photocatalytic activity of P-CN. Once constructing the heterojunction by calcinating the mixture of P-CN and Rh-STO, all the heterostructured P-CN/Rh-STO showed superior performances in contrast to that of P-CN, indicating that the formation of heterojunction between P-CN and Rh-STO can promote the charge separation and thus enhance the photocatalytic activity of P-CN/Rh-STO (Figure 6a). With the increase of the amount of Rh-STO, the photocatalytic activity of P-CN/Rh-STO gradually increased and then decreased. The optimized P-CN/Rh-STO-20 obtained the highest activity of 4.451 ± 0.093 mmol g^−1^ h^−1^ (Figure 6b), which 3.91 times and 8.56 times those of P-CN (1.138 ± 0.015 mmol g^−1^ h^−1^) and Rh-STO (0.520 ± 0.025 mmol g^−1^ h^−1^), respectively. As discussed before, P-CN/Rh-STO-20 with type II band alignment structure possessed extended visible light absorption in contrast to that of P-CN. Moreover, the charge separation and transfer in P-CN/Rh-STO-20 were effectively promoted by the heterojunction between P-CN and Rh-STO. Therefore, the enhanced photocatalytic performance of P-CN/Rh-STO-20 should be attributed to the extended light absorption and the enhanced charge separation by the heterojunction. Moreover, it can be seen in Table S3 that the P-CN/Rh-STO-20 in this work possessed a superior photocatalytic activity towards the recently reported C_3_N_4_-based photocatalysts. In addition, we also tested the stability of the catalyst, as shown in Figure 6c. After three cycles, it can be seen that P-CN/Rh-STO-20 showed good durability and higher photocatalytic activity in contrast to those of P-CN and Rh-STO. The slight decrease in activity of P-CN/Rh-STO-20 after three cycles might be a result of the depletion of the sacrificial reagents or the generation of byproducts resulted from the oxidation of sacrificial reagents during the photocatalytic process. In addition, an experiment of photodeposition of Pt nanoparticles was conducted to confirm the formation of type II heterojunctions. Briefly, the sample of P-CN/Rh-STO-20 and a calculated amount of H_2_PtCl_6_ (1 wt% Pt) were mixed in a solution containing 10 vol% TEOA, and irradiated by a 300 W Xe lamp coupled with a 400 nm cutoff filter for 1 h under magnetic stir. After the reaction, the sample was washed and centrifuged several times for the TEM characterization. As shown in Figure S4, it was clear that Pt nanoparticles were photo-deposited on the surface of P-CN, and no Pt nanoparticles were found on Rh-STO, confirming that H_2_PtCl_6_ was reduced by the photogenerated electrons in P-CN rather than that in Rh-STO, and thus revealing that P-CN/Rh-STO-20 was a type II heterojunction, not the Z-scheme system. Figure 6d shows the charge transfer mechanism in P-CN/Rh-STO-20 during the photocatalytic process. In the type-II heterostructured P-CN/Rh-STO-20, the conduction band bottom of Rh-STO (−1.89 eV) was more negative than that of P-CN (−0.41 eV), while the valence band top of P-CN (2.29 eV) was more positive than that of Rh-STO (0.64 eV). When the P-CN/Rh-STO−20 was irradiated by visible light, the photogenerated electrons excited to the conduction band of Rh-STO would flow across the heterojunction interface to the conduction band of P-CN, and the photogenerated holes would flow from the valence band of P-CN to the valence band of Rh-STO. Therefore, the photogenerated electrons and holes will gather on two different semiconductors to realize spatial separation and participate in reduction and oxidation reactions, respectively, thus improving the photocatalytic hydrogen production performance of the catalyst, which was consistent with the experimental results.

## 4. Conclusions

In this work, a type-II heterostructured photocatalyst was constructed by compositing phosphorus-doped g-C_3_N_4_ (P-CN) and Rh-doped SrTiO_3_ (Rh-STO) via a thermal calcination treatment. During the calcination process, the in situ generated P element, which resulted from the decomposition of P-CN, can interact with Rh-STO, and thus lead to the formation of heterojunction between P-CN and Rh-STO. The P-CN/Rh-STO-20 sample exhibited obtained the highest activity of 4.451 ± 0.093 mmol g^−1^ h^−1^, which was 3.91 times and 8.56 times that of P-CN (1.138 ± 0.015 mmol g^−1^ h^−1^) and Rh-STO (0.520 ± 0.025 mmol g^−1^ h^−1^), respectively. It was revealed by the results of UV–visible light absorption and XPS that the heterostructured P-CN/Rh-STO-20 possessed a type II band alignment, greatly promoting the charge separation during the photocatalytic process, and thus leading to the superior activity of P-CN/Rh-STO-20 in contrast to those of P-CN and Rh-STO.

## Data Availability

The data presented in this article are available on request from the corresponding author.

## Supplementary Materials

The following supporting information can be downloaded at: https://www.mdpi.com/article/10.3390/nano12244428/s1, The detail of characterization, photocatalytic activity measurements, photoelectrochemical measurements; Table S1: ICP-MS analysis of P-CN/Rh-STO-20 and P-CN; Table S2: Fluorescence lifetime of P-CN and P-CN/Rh-STO-20; Table S3: The comparison of the activity with recently reported C_3_N_4_-based photocatalysts; Figure S1: The TGA analysis of Rh-STO-20; Figure S2: The TGA analysis of P-CN. Figure S3: The TGA analysis of P-CN/Rh-STO-20; Figure S4: TEM image of 1wt% Pt loaded P-CN/Rh-STO-20.
