# Supplementary material for: The Construction of Phosphorus-Doped g-C3N4/Rh-Doped SrTiO3 with Type-II Band Alignment for Efficient Photocatalytic Hydrogen Evolution
Source: Nanomaterials (Basel). 2022 Dec 12;12(24):4428. doi: 10.3390/nano12244428 (PMC9782634; doi:10.3390/nano12244428)
Supplement: Supplementary file 1 [file nanomaterials-12-04428-s001.zip › nanomaterials-2065936-supplementary.pdf]

nanomaterials

MDPI

Supplementary Materials

The Construction of Phosphorus-Doped g-C_3N_4/Rh-Doped $SrTiO_3$ with Type-II Band Alignment for Efficient Photocatalytic Hydrogen Evolution

Bin Wang [1,2,3,*], Peng Li [1], Hanjing Hao [1], Huijie He [1], Hairui Cai[1], Fanfan Shang [1], Bei An [1], Xiaoqian Li [1] and Shengchun Yang [1,2,3,*]

[1] MOE Key Laboratory for Non-Equilibrium Synthesis and Modulation of Condensed Matter, Key Laboratory of Shaanxi for Advanced Materials and Mesoscopic Physics, State Key Laboratory for Mechanical Behavior of Materials, School of Physics, Xi'an Jiaotong University, No. 28 West Xianning Road, Xi'an 710049, China

[2] National Innovation Platform (Center) for Industry-Education Integration of Energy Storage Technology, Xi'an Jiaotong University, No. 28 West Xianning Road, Xi'an 710049, China

[3] Shaanxi Collaborative Innovation Center for Hydrogen Fuel Cell Performance Improvement, Xi'an Jiaotong University, No. 28 West Xianning Road, Xi'an 710049, China

* Correspondence: bin_wang@xjtu.edu.cn (B.W.); ysch1209@mail.xjtu.edu.cn (S.Y.)

1. Characterization

The X-ray diffraction (XRD) patterns were obtained from a PANalytical X'pert MPD Pro diffractometer operated at 40 kV and 40 mA using Ni-filtered Cu Kα irradiation (Wavelength 1.5406 Å). UV-vis absorption spectra (UV-vis) were measured on a HITACHI U4100 instrument equipped with a labsphere diffuse reflectance accessory using $BaSO_4$ as the reference. The transmission electron microscopy (TEM) images, high-resolution TEM (HRTEM) images, and the high angle annular dark field-scanning transmission electron microscopy (HAADF-STEM) images were obtained from a JEOL JEM-F200 transmission electron microscope at an accelerating voltage of 200 kV. An OXFORDMAX-80 energy-dispersive X-ray detector (EDX) which was mounted in the above TEM was used to conduct elemental analysis. X-ray photoelectron spectroscopy (XPS) measurements were conducted on a Thermo Fisher ESCALAB Xi+ with monochromatic Al Kα radiation (hν = 1486.69 eV) and with the pressure of sample analysis chamber under high vacuum $<5\times10^{-10}$ mbar. All binding energies were referenced to the C 1s peak at 284.8 eV. Fourier transform infrared (FTIR) spectra were recorded on a Bruker Vextex 70 FTIR spectrometer using the KBr pellet technique. The total Pt and Co contents in the real sample were analyzed by Inductively Coupled Plasma Mass Spectrometer (ICP-MS) (NexIONTM 350D, PerkinElmer, USA). The PL spectra were carried out at room temperature using an Edinburgh FLS9 fluorescence spectrophotometer. In detail, PL emission spectra were obtained at an excitation wavelength of 375 nm. The lifetime of carriers in the catalyst were fitted based on the following equation:

$$\text{Fit} = A + B_1 \exp\left(-\frac{t}{\tau_1}\right) + B_2 \exp\left(-\frac{t}{\tau_2}\right) + B_3 \exp\left(-\frac{t}{\tau_3}\right) \quad (1)$$

Where: Fit -- fitting function; A、B_1、B_2、B_3 - decay constant; t -- time; τ_1、τ_2、τ_3 - decay parameters.

Based on the above fitting curve, the average life (τ) of carriers in the catalyst through the following equation：

$$\tau = \frac{B_1\tau_1^2+B_2\tau_2^2+B_3\tau_3^2}{B_1\tau_1+B_2\tau_2+B_3\tau_3} \quad (2)$$

Where: τ -- average life span; B_1、B_2、B_3 -- decay constant; τ_1、τ_2、τ_3 -- decay parameters.

2. Photoelectrochemical measurements

The working electrode was prepared according to our previous work. Briefly, 5 mg catalyst was dispersed in 1mL isopropanol with 25 uL nafion (0.5 %) solution to form a suspension solution by sonication. Then, 5 uL catalyst solution was loaded onto the L-type glassy carbon electrode. The catalyst-coated area was fixed *ca.* 0.25 $cm^{2.}$ Finally, the deposited electrode was dried in a vacuum oven at room temperature overnight. Photoelectrochemical and electrochemical measurements were carried out in an electrochemical workstation (CHI760 Instruments) based on a conventional three-electrode system, where a sample-coated L-type Glassy Carbon Electrode, platinum foil (2×2cm) and a saturated calomel electrode (SCE) were used as the working electrode, counter electrode and reference electrode, respectively. The electrolyte solution was 0.5 M Na_2SO_4 solution containing 10 vol % TEOA.

Table S1. ICP-MS analysis of P-CN/Rh-STO-20 and P-CN.

catalyst	C_P(mg·L^{-1})	P wt%(*vs.* P-CN/Rh-STO-20)	P wt%(*vs.* P-CN)
P-CN/Rh-STO-20	9.42	1.88%	2.38%
P-CN	10.50	/	2.10%

Table S2. Fluorescence lifetime of P-CN and P-CN/Rh-STO-20.

**catalyst**	**B_1**	**τ_1(ns)**	**B_2**	**τ_2(ns)**	**B_3**	**τ_3(ns)**	**τ(ns)**
P-CN	23265	0.97	9559	3.55	869	16.11	5.22
P-CN/Rh-STO-20	21337	1.04	9862	3.72	922	16.91	5.68

Table S3. the comparison of the activity with recently reported C_3N_4-based photocatalysts.

Samples	Sacrificial reagents	wavelength	Rate of H_2 evolution (mol g^{-1} h^{-1})	References
P-CN/Rh-STO-20	10 vol% TEOA solution	≥ 400 nm	4.451	This work
ZnS/g-C_3N_4	0.35 M Na_2S and 0.25 M Na_2SO_3 solution	≥ 420 nm	0.71368	Applied Catalysis B: Environmental, 2018, 229: 41-51.
NiCoP/g-C_3N_4	15 vol% TEOA solution	≥ 420 nm	1.06711	ChemistrySelect, 2021, 6(24): 5967-5974.
$LaFeO_3$/g-C_3N_4 nanosheet-graphene heterojunction	20 vol% TEOA solution	≥ 420 nm	1.3265	Journal of Alloys and Compounds, 2022, 890: 161850.
$SnFe_2O_4$/g-C_3N_4	10 vol% TEOA solution	≥ 420 nm	2.65	Applied Surface Science, 2020, 506: 144939.
CdS/PdAg/g-C_3N_4	10 vol% TEOA solution	≥ 400 nm	3.0983	Applied Catalysis B: Environmental, 2021, 281: 119509.
Co_2PO_4OH/g-C_3N_4	10 vol% methanol solution	direct solar light	0.254	International Journal of Hydrogen Energy, 2020, 45(13): 7562-7573.
B-TiO_2/g-C_3N_4	10 vol% TEOA solution	Full wavelength	0.80897	Applied Catalysis B: Environmental, 2019, 242: 92-99.
$ZnIn_2S_4$/S doped g-C_3N_4	10 vol% TEOA solution	≥ 420 nm	1.64	Journal of Alloys and Compounds, 2022, 924: 166569.
g-C_3N_4/Ag_2CrO_4	25 vol% methanol solution	≥ 420 nm	0.9021	Scientific reports, 2018, 8(1): 1-12.
g-C_3N_4/WO_3	10 vol% TEOA solution	Full wavelength	3.12	Applied Catalysis B: Environmental, 2017, 219: 693-704.
$MnIn_2S_4$/g-C_3N_4	0.35 M Na_2S and 0.25 M Na_2SO_3 solution	≥ 400 nm	0.2008	Chemical Engineering Journal, 2019, 359: 244-253.
$CuInS_2$@C_3N_4	10 vol% triethanolamine along with 0.25 M Na_2S and 0.2 M Na_2SO_3 solutions	≥ 420 nm	0.373	Chinese Journal of Catalysis, 2020, 41(1): 122-130.

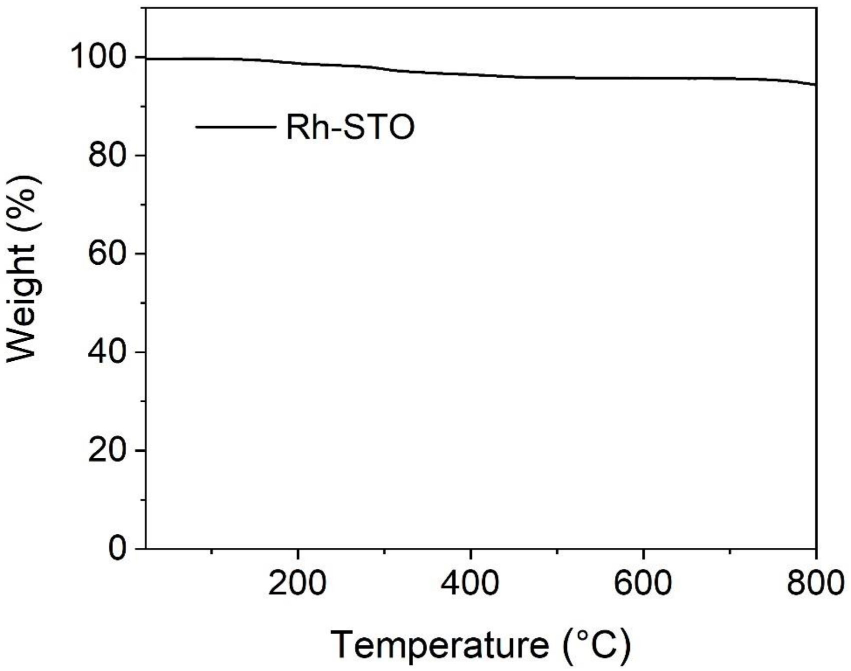

Figure S1. The TGA analysis of Rh-STO.

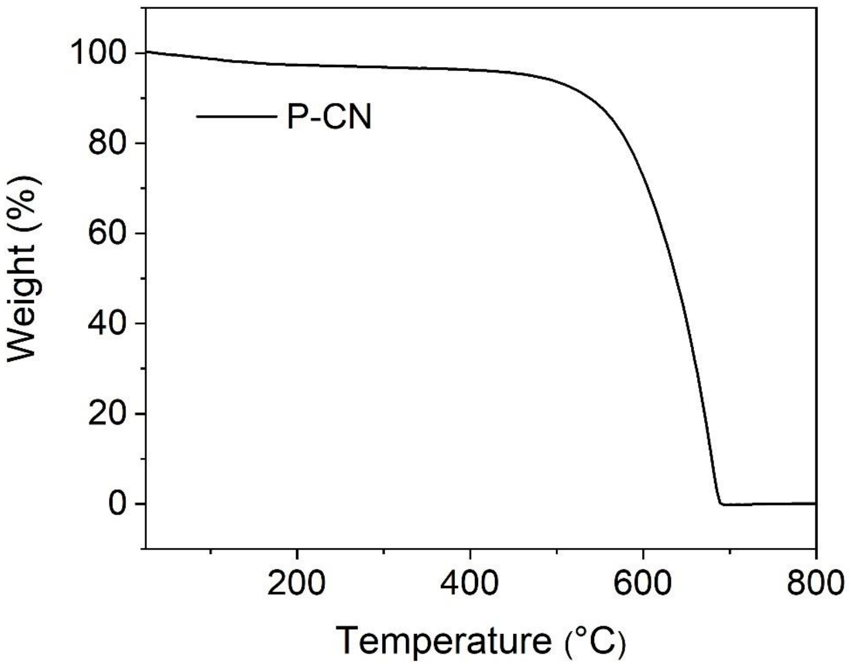

Figure S2. The TGA analysis of P-CN.

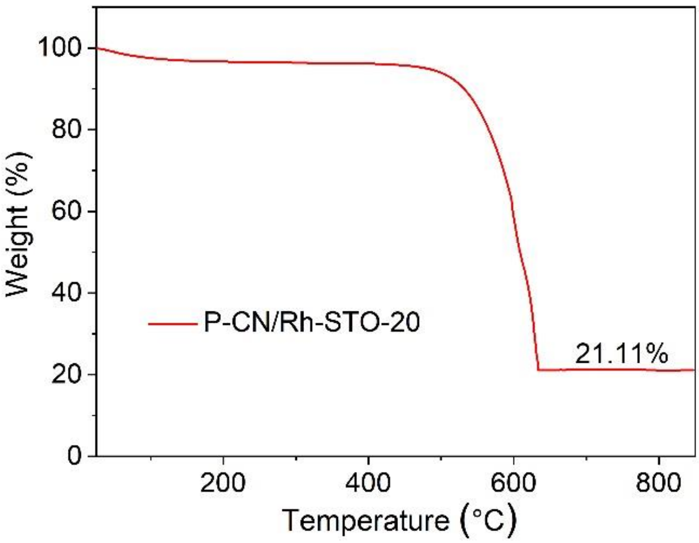

Figure S3. The TGA analysis of P-CN/Rh-STO-20.

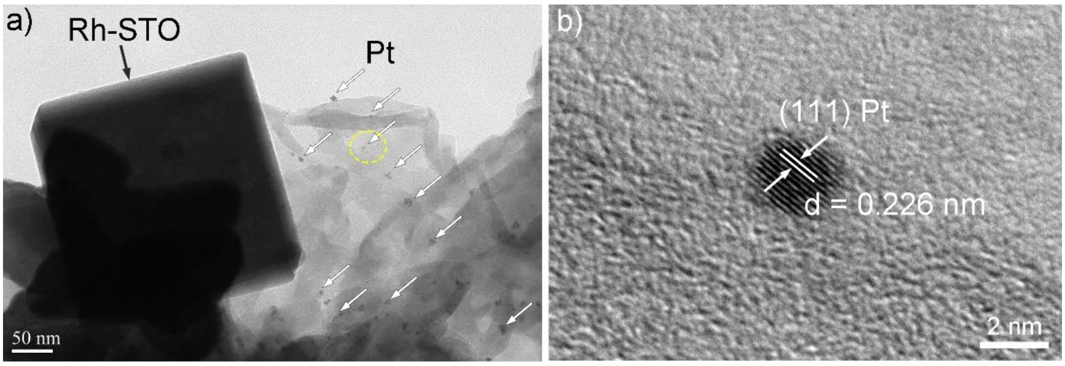

Figure S4. (a) TEM image of 1wt% Pt loaded P-CN/Rh-STO-20; (b) HRTEM image of Pt nanoparticles recorded from the yellow dotted circle in (a). The white arrows indicated the photo-deposited Pt nanoparticles on P-CN/Rh-STO-20.
